# Genetic architecture of fresh-market tomato yield

**DOI:** 10.1186/s12870-022-04018-5

**Published:** 2023-01-09

**Authors:** Prashant Bhandari, Juhee Kim, Tong Geon Lee

**Affiliations:** 1 Horticultural Sciences Department, University of Florida, Gainesville, FL 32611 USA; 2 Gulf Coast Research and Education Center, University of Florida, Wimauma, FL 33598 USA; 3 Plant Breeders Working Group, University of Florida, Gainesville, FL 32611 USA; 4 Plant Molecular and Cellular Biology Graduate Program, University of Florida, Gainesville, FL 32611 USA; 5 Bayer, Chesterfield, MO 63017 USA

**Keywords:** Tomato, Genetic improvement, Yield, Genetic mapping, Genomic estimated breeding value, Genomic selection

## Abstract

**Background:**

The fresh-market tomato (*Solanum lycopersicum*) is bred for direct consumption and is selected for a high yield of large fruits. To understand the genetic variations (distinct types of DNA sequence polymorphism) that influence the yield, we collected the phenotypic variations in the yields of total fruit, extra-large-sized fruit, small-sized fruit, or red-colored fruit from 68 core inbred contemporary U.S. fresh-market tomatoes for three consecutive years and the genomic information in 8,289,741 single nucleotide polymorphism (SNP) positions from the whole-genome resequencing of these tomatoes.

**Results:**

Genome-wide association (GWA) mapping using the SNP data with or without SNP filtering steps using the regularization methods, validated with quantitative trait loci (QTL) linkage mapping, identified 18 significant association signals for traits evaluated. Among them, 10 of which were not located within genomic regions previously identified as being associated with fruit size/shape. When mapping-driven association signals [558 SNPs associated with 28 yield (component) traits] were used to calculate genomic estimated breeding values (GEBVs) of evaluated traits, the prediction accuracies of the extra-large-sized fruit and small-sized fruit yields were higher than those of the total and red-colored fruit yields, as we tested the generated breeding values in inbred tomatoes and F_2_ populations. Improved accuracy in GEBV calculation of evaluated traits was achieved by using 364 SNPs identified using the regularization methods.

**Conclusions:**

Together, these results provide an understanding of the genetic variations underlying the heritable phenotypic variability in yield in contemporary tomato breeding and the information necessary for improving such economically important and complex quantitative trait through breeding.

**Supplementary Information:**

The online version contains supplementary material available at 10.1186/s12870-022-04018-5.

## Background

Tomato (*Solanum lycopersicum*) is the most valuable fruit crop worldwide [1] and it provides essential micronutrients [2]. The fresh-market tomato is one of the two most widely consumed types of contemporary tomatoes, and it is bred for direct consumption [2]. The other is the processing tomato, destined for processed foods such as ketchup.

Yield is the top priority for plant breeding and improvement programs, and it is a quantitative trait. The fresh-market tomato is selected for a high yield of large fruits (e.g., extra-large-sized fruit (fruit size > 6.985 cm in diameter) [3]), especially in the U.S., to satisfy market demands and production systems for over a century [4]. This was achieved by mating selected breeding lines. Reintroduction of genetic diversity through inter- (disease resistance such as Fusarium wilt resistance in fresh-market tomatoes [5]) or intra- (plant architecture such as brachytic trait in fresh-market tomatoes [6]) species crosses, similar to that in other breeding programs (e.g., [7]), has been applied to the fresh-market tomato [5, 8, 9]. The *GLOBE* gene, which affects fruit shape, was recently identified from the U.S. fresh-market tomatoes [10]; the gene has the potential to increase a fruit size in the form of F_1_ hybrid (S.F. Hutton, personal communication). Therefore, the frequencies of beneficial alleles could have simultaneously increased across relevant breeding lines, regardless of whether such alleles have been readily identified or not.

The identification of genetic loci corresponding to specific traits is one of the first steps to build a scientific basis for plant genetics and breeding. Both linkage and genome-wide association (GWA) mappings are used to map tomato traits (e.g., 73 quantitative trait loci (QTL) known to control fruit size (weight)/shape [11], sugar content [12], flavor [13]). However, the genetic architecture (characteristics of DNA sequence variations responsible for traits) of yield [fruit weight (size) × fruit number] in the contemporary fresh-market tomato remains largely unknown. There are at least four underlying reasons; first, studies have often focused on individual components of yield (e.g., fruit weight) rather than the yield itself (*SICLV3* [14]; reviewed in Ariizumi et al. [15], Zsögön et al. [16], Xia et al. [17]); in addition, the phenotypic variations are not fully examined even for these individual components (only a fraction of a plant’s total fruits is harvested [10, 18–20]). Second, the lack of the awareness and appreciation for different fruit market classes of contemporary tomatoes. There is a high demand for fruits that are large (i.e., extra-large-sized fruit) in the U.S. fresh-market tomato class; therefore, the yield of such large-sized fruit is considered important by fresh-market tomato breeders [21, 22]. Third, most of the mapping studies have focused on general genetic studies using the genetic background of model tomatoes for research (e.g., Micro-Tom, M82) and/or have included none or a few of the contemporary fresh-market tomato germplasm (discussion in Bhandari et al. [23]). Several studies used a population derived from M82 (*S. lycopersicum*; domesticated model tomato and not a contemporary fresh-market tomato) × LA716 (*S. pennellii*; wild tomato) [24–26]. The *SUCR* gene found in the wild tomato *S. chmielewskii* was tested in a processing tomato [27]. Finally, the analysis of phenotypic variation in tomato has often focused on differences between species (domesticated vs. wild tomatoes); phenotypic/genotypic variance underlying much of the variation in evaluated traits was found to be already fixed in most contemporary fresh-market tomato germplasm (further discussion in the following section).

Fruit size (weight) is an important trait in fruit crops because it influences yield [28]; many loci responsible for fruit size (weight) variations in tomato are relatively well identified. The fruit size (weight) loci identified through a comparison of domesticated and wild tomatoes (e.g., *FASCIATED* [29], *FW2.2* [30], *FW3.2* [5, 31, 32], *LOCULE-NUMBER* [33]) and the shape loci (e.g., *OVATE* [34], *SUN* [35]) contribute to the majority of the tomato fruit size variations. Importantly, a few combinations of alleles at such known loci are found in several contemporary fresh-market tomato germplasm, suggesting that many of these loci could have been already fixed for desirable alleles in their germplasm [22, 36–38]. These alleles could have made an important contribution towards the genetic background, during fruit domestication and/or historical improvement in tomato (*S. lycopersicum*), resulting in an overall increased size, compared to that in the closest wild species (*S. pimpinellifolium*) [39]. However, little is known about the genetic variations responsible for the current heritable phenotypic variability in yield in contemporary tomato breeding. Genetic engineering (e.g., gene knockouts or overexpression strategies) of candidate genes or gene-regulatory regions increases fruit size [14, 40] in a few model tomato backgrounds; this enables generating new genetic diversity. However, consumer acceptance of genetically engineered tomatoes is a complex issue because the general public’s response to these technologies is variable. Therefore, the genetic architecture of yield in the contemporary fresh-market tomato needs to be explored to accelerate breeding efforts.

Sparse models can be applied in quantifying collinear phenotypes and genotypes (dependent and independent variables, respectively) [41]. Regularization methods [minimax concave penalty (MCP) [42], least absolute shrinkage and selection operator (LASSO) [43]] are used to introduce sparsity by pruning overfit models [43, 44]; they are adopted in both linkage and GWA mappings [45–52] and genomic selection (GS) [53]] for feature selection. Partitioning phenotypic variations that are controlled by genotype, population structure, kinship, and/or other covariates in a population are a challenge, especially when the traits are quantitative, bred for a particular environment, or have undergone a strong selection pressure [4]. In such cases, regularization methods can be adapted to select features (i.e., variables) or estimate loci effects.

In this study, we aimed to map whole-genome sequencing (WGS)-based single nucleotide polymorphism (SNP)-trait associations for total yield (hereafter, *Y*) and three key contemporary fresh-market tomato traits; we focused on the yields of extra-large-sized fruit (*XY*), any fruit smaller than medium size [hereafter referred to as small-sized fruit (*SY*)], or red-colored fruit regardless of size (*RY*), in a group of 68 core inbred contemporary U.S. fresh-market tomatoes, for elucidating the genetic basis of these traits. GWA mapping using the SNP data with or without SNP filtering steps using the regularization methods, validated with QTL linkage mapping, was used to identify association signals for the evaluated traits. In fresh-market tomato breeding programs, emphasis was on a high yield of large fruit (*XY*); phenotyping of small fruits (*SY*) was ignored. Therefore, the genetic mechanism by which *SY* contributes to *Y* and/or *XY* remains unknown. Fruit coloring (changing color from green to red) is an important trait in fresh-market tomato. The fruit markets and the fruit color at harvest are tightly linked, which directs breeding aims (green color fruits or red ones for the food supply or the retail, respectively). Fruit coloring is also an essential phenotype for postharvest handling of fruits including shipping and long-term storage. This study reports the first genomic estimated breeding values (GEBVs) generated for the yield of fresh-market tomato. GS [54] has considerable potential for improving complex traits, such as yield, which is controlled by QTL with small effects in applied breeding program. There are GS studies in tomato [55–61]. However, there is no prior GS study for the yield of fresh-market tomato. To infer the effects of associations identified by GWA/QTL linkage mappings on estimating the yield of fresh-market tomato, we compared the prediction accuracies among four different SNP sets, three sets from mappings or regularization methods-driven SNPs and a set from SNPs evenly distributed across the tomato genome. We trained and tested the generated breeding values in both inbred tomatoes and F_2_ populations.

## Results

### Variation and correlation for fresh-market tomato yields

**Fig. 1:**
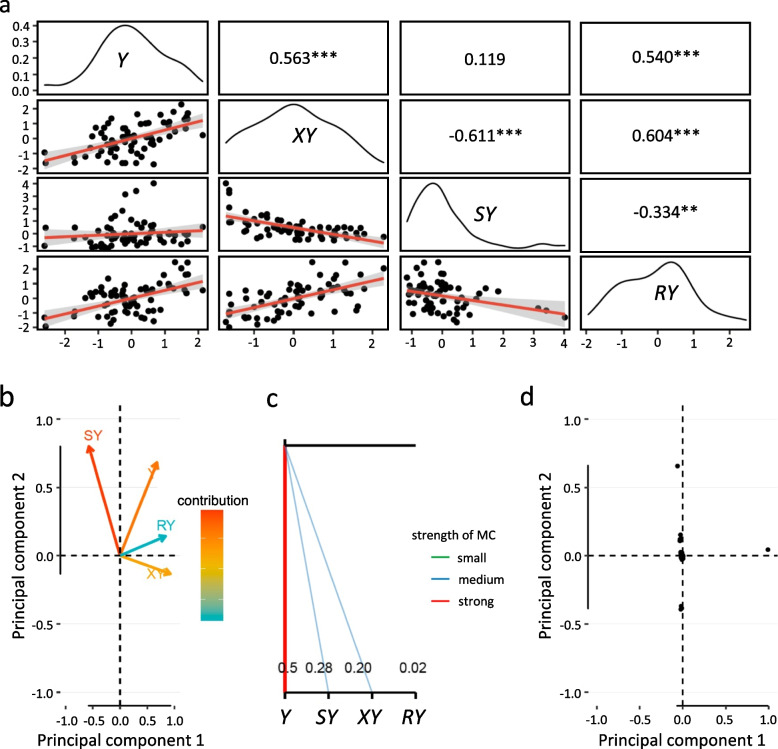
Variation and correlation in fresh-market tomato yields. **a** The distribution of BLUPs for phenotypic values of four traits in 68 core inbred contemporary fresh-market tomatoes (inbred tomato set) are indicated by the black lines [*Y*, the total yield; *XY*, the yield of extra-large-sized fruit; *SY*, the yield of any fruit smaller than medium size; *RY*, the yield of red-colored fruit regardless of size (**a** through **c**)]. The numerical correlation coefficients (*r* value) with statistical significance (^**^p < 0.01, ^***^p < 0.001) and scatter plots of correlations between two traits are shown in the upper and lower panels, respectively. The red lines in the scatter plots represent the correlation trends; the gray areas indicate standard errors. **b** Loading plot of principal component (PC)1 and PC2 of *Y*, *XY*, *SY*, and *RY* traits. **c** Multi-collinearity analysis. The strength of collinearity is indicated by the darkness and thickness of line. **d** Principal component analysis (PCA) of SNP variations in the inbred tomato set

To examine the phenotypic variation in yield for the 68 core contemporary fresh-market tomatoes, we measured phenotypic values of 64,125 fruits totaling 4786 kg in weight for three successive years. Phenotypic values of four traits, the yields of total fruit (*Y*), extra-large fruit (*XY*), small-sized fruit (*SY*), or red-colored fruit (*RY*), ranged from 1.6 (mean of 3 years) to 7.3 kg for *Y*, 0.1 to 4.3 kg for *XY*, 0.2 to 5.5 kg for *SY*, and 0.1 to 3.8 kg for *RY*. A normal distribution for *Y*, *XY*, and *RY* (except *SY*) was observed for each of the 3 years (Additional file 1). The phenotypic data was converted to BLUPs to reduce environmental deviation in further analysis as suggested by Piepho et al. [62] (Additional file 2). Pearson’s correlation among the traits indicated that high degree of positive correlations were observed between *Y* and *XY* (*r* = 0.56) and between *Y* and *RY* (0.54) or between *XY* and *RY* (0.60) (Fig. 1a; upper panel). Interestingly, there was a strong negative correlation between *XY* and *SY* (− 0.61); there was no correlation between *Y* and *SY*. Relatively high heritability was estimated for *XY* and *SY* at 0.71 and 0.86, respectively. Moderate (0.57) and low (0.29) heritability was estimated for *Y* and *RY*, respectively. We performed PCA of four traits that were focused. PC1 and PC2 explained 58 and 26% of the trait variance, respectively (Fig. 1b). Both *XY* and *RY* showed high positive correlations with PC1, while *SY* showed negative correlation with PC1. PC2 explained the majority of *SY* variation, whereas both PC1 and PC2 explained *Y* variation. In addition, *Y* is responsible for the collinearity between *Y* and *SY*, and *Y* and *XY* (Fig. 1c). There was no such collinearity between *Y* and *RY*.

**Fig. 2:**
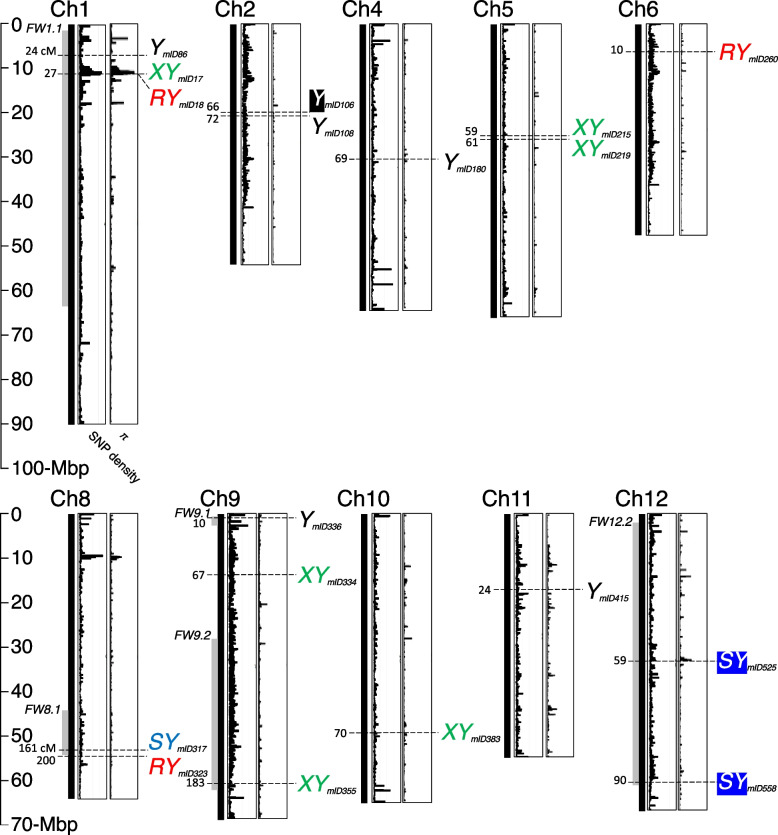
Chromosomal distribution of DNA sequence variant-trait associations for fresh-market tomato yields. The positions of significant common genome-wide association (GWA) signals (derived from both the filtered SNP set and regularization SNP set) are indicated by the symbols of corresponding traits (*Y*, the total yield; *XY*, the yield of extra-large-sized fruit; *SY*, the yield of any fruit smaller than medium size; *RY*, the yield of red-colored fruit regardless of size). Mapping IDs are indicated immediately below to the right of the symbol of GWA. Black and blue rectangles on chromosomes 2 (Ch2) and 12 (Ch12) highlight the significant common association signals that fall within the intervals of linkage mapping in this study. Numbers with dashed horizontal lines indicate estimated genetic positions (cM) of association signals based on the fresh-market tomato genetic map. The approximate intervals of previously mapped fruit size (weight) loci with a prefix *FW* are depicted using gray vertical lines. For each chromosome, SNP density (the range of the *Y*-axis 0 to 400 SNPs per 10-kbp) and nucleotide diversity (*π*) (the range of the *Y*-axis 0 to 0.08) plots are provided. A detailed dataset of this figure with the Manhattan plots of SNPs associated with traits and the quantile-quantile plots for association mapping can be found in Additional files 5, 6, and 7

To obtain genomic information of these tomatoes, we used the whole-genome resequencing-driven nucleotide sequences; we identified 8,289,741 SNP sites, or approximately 1 SNP/kbp, against the tomato reference genome sequence SL4.0 [63] (Fig. 2; Additional file 3). We validated genotype calls by using the deep (over 100 × genome coverage) sequencing data of a tomato material Fla. 8814 (Table S1 in Lee [64]). There was a high consistency (> 97%) in the SNP calls of Fla. 8814 between Lee, 2022 and this study. Using the filtered SNP set, we estimated nucleotide diversity (*π*) to be 0.0003 ± 0.0015 (s.d.) (Fig. 2; Additional file 3). Distinct genomic regions with relatively high *π* values were observed, which indicate evidence of crossover during natural or artificial crossing and selection (e.g., known wild tomato (*S. pennellii*) introgression near 60-Mbp on chromosome 7; [64, 65]) among the tomato materials. The measurement of inbreeding coefficient (*F*) shows that most of tomatoes show positive values, ranging between 0 to 0.5 (Additional file 4). However, multiple tomatoes had an overall whole genome-wide negative *F* values, predominantly found along with chromosomes 1, 3, 8, and 10. PC1 and PC2 from PCA of SNP variations identified five clines (Fig. 1d).

### Genetic associations between DNA variants and fresh-market tomato yields

We conducted two GWA mappings, one using the filtered SNP set and the other using the regularization SNP set. In the first GWA mapping, a total of 85 association signals (27, 14, 29, and 15 for *Y*, *XY*, *SY*, and *RY*, respectively) were detected on at least one of the six models (Additional files 5 and 6). Among these 85 signals, a signal (mapping ID *mID526*) had significant effects on both *Y* and *XY* traits. Subsequently, 42 association signals were identified in the second GWA mapping: 14, 13, 5, and 10 for *Y*, *XY*, *SY*, and *RY*, respectively (Additional files 6 and 7). Importantly, 18 association signals (6, 6, 3, and 3 for *Y*, *XY*, *SY*, and *RY*, respectively) were common between the two GWA mappings (hereafter referred to as common association signals; Fig. 2).

Several common associations were found in/near genomic regions with relatively high nucleotide diversity in these contemporary fresh-market tomatoes. Three common associations (*mID17*, *mID18*, and *mID86* associated with *XY*, *RY*, and *Y*, respectively) were located within the multi-megabases interval (within 20% of the chromosome length) with high nucleotide diversity on chromosome 1, which overlaps with the known fruit size locus *FW1.1* [11, 39, 66] (Fig. 2). A limited recombination density between *mID17* and *mID18* indicates the possibility that these association signals were originated from the same locus. The significant allelic effects on traits were not correlated with distinct clines observed in PCA (Additional file 8). For example, the allelic effect of the association *mID86* on *Y* [homozygous genotype (TT) with higher *Y* vs. heterozygote (AT) with lower *Y*] was not correlated with five distinct clines. Further, this chromosome 1 *Y* SNP [i.e., the homozygous genotype (TT) at *mID86*] had a significant positive association with *XY* (ANOVA at *p* < 0.05; Additional file 9). Based on the published Heinz 1706 processing tomato genome [67] and its genome annotation ITAG4.0 [63], the SNP was in the upstream intergenic region of *Solyc01g011310*, which encodes an aspartic proteinase-like protein 2 involved in plant growth and development [68].

A common association (*mID317*) fell within the known fruit size locus *FW8.1* [11, 69] on chromosome 8 and had a significant effect on *SY* (Fig. 2); one possible positional candidate gene (*Solyc08g062940*), which is located approximately 170-kbp downstream of the association signal, encodes an IQ calmodulin-binding motif containing protein in the Phytozome database (https://phytozome-next.jgi.doe.gov) and is a member of the *SUN* gene family [70] whose member is associated with fruit development [35]. Another common association (*mID336*) with *Y* was located close to the telomere (within 5% of the chromosome length) of chromosome 9, which overlaps with the known fruit size locus *FW9.1* [11, 39, 71] (Fig. 2). Another known fruit size locus *FW9.2* spans > 30-Mbp on chromosome 9 [11, 72, 73]. The homozygous genotype (CC) of a common association (*mID355*), which fell within the interval of *FW 9.2*, had a significant positive effect on *XY* (Fig. 2). Contrasting associations between the two different traits *SY* and *RY* and the SNP in the *FW9.2* mapping interval were determined using ANOVA: *SY* and *RY* exhibited lower and higher BLUP values, respectively, with the homozygous CC genotype compared to that in the tomatoes carrying the other allele (Additional file 9).

We identified 10 common association signals that do not overlap with previously mapped fruit size (weight)/shape loci (Fig. 2); most of these previously mapped loci spanned wide genomic areas across 12 chromosomes of tomato. Four common associations had significant effects on *XY*. The homozygous genotype (AA) at the one of two common associations on chromosome 5 (*mID219*) had a significant positive effect on *XY*. Interestingly, the same allele type showed significant associations (ANOVA at *p* < 0.05) with two other traits, similar to the *mID355* association: high *Y* and low *SY* (Additional file 9). Another positive *XY* association (*mID334*) with the homozygous genotype TT was found in chromosome 9. The identical homozygous genotype showed contrasting associations between the two other traits: low *SY* and high *RY*, as determined using ANOVA (Additional file 8). The SNP is located at ~ 2-kbp upstream of a retrovirus-related Pol polyprotein from transposon TNT 1–94 (*Solyc09g018237*) and is a member of a gene family predicted to participate in thermotolerance response in tomato [60]. Additionally, another common association (*mID415*) occurred on chromosome 11. The heterozygous genotype at this position was positively associated with three traits, *Y* determined using GWA mappings, and *XY* and *RY*, using ANOVA. The association was flanked by two previously identified GWA signals for fruit traits (i.e., fruit fasciation and asymmetry) in the European traditional tomato [74]; all three of these associations were placed into a 45-kbp interval.

For *RY*, a chromosome 8 association (*mID323*) was identified through GWA mappings (Fig. 2). An allele identical with the high *RY* association signal had positive associations with *Y* and *XY* (ANOVA at *p* < 0.05) (Additional file 9). This chromosome 8 *RY* SNP is located approximately 1.2-Mbp downstream of the *FW8.1* locus [11, 69] and < 1-Mbp upstream of the previously identified association signal for tomato fruit weight [19].

Fifty-two significant linkage mapping signals (13, 4, 26, and 9 for *Y*, *XY*, *SY*, and *RY*, respectively) were identified in the QTL linkage mapping of four traits in the F_2_ populations of contemporary fresh-market tomatoes (Additional file 6). We examined overlaps between the common association signals obtained from GWA mappings and the putative regions identified through the QTL linkage mapping. We found three common association signals (one with *Y* on chromosome 2 and two with *SY* on chromosome 12), which overlap with the linkage mapping (Fig. 2). Given the *FW12.2* spans > 57-Mbp on the SL4.0 version of the reference genome assembly [11], both chromosome 12 *SY* associations (*mID525* and *mID558*) fell within the interval of *FW12.2*. Interestingly, a substantial recombination density (> 30 cM) separated the two *SY* associations when the positions of these two associations were projected onto the fresh-market tomato genetic map. The homozygous genotype (AA) at the *mID525* was associated with a significant effect on low *SY*; this was validated in an additional experiment using an F_2_ population D (Additional file 10). The SNP had a significant association with high *XY* as determined using ANOVA (Additional file 9). A CST complex subunit CTC1 (*Solyc12g062480*) is found < 40-kbp downstream of the *mID525* association signal and is related to plant morphology [75]. Additionally, the presence and absence of the several sequence variants [a structural variant (77 bp deletion starting at position 32,893,595 bp on chromosome 12), an 8 bp insertion (between positions 32,898,595 bp and 32,893,596 bp), and two SNPs on 32,906,405 bp or 32,906,415 bp] encompassing part of the *Solyc12g062480* gene were found near the association in the fresh-market tomato lines, Fla. 8924 and Fla. 8814, with available long-read sequence data (Fla. 8924 from Alonge et al. [5]; Fla. 8814 from Lee [64]). Such sequence variants were present in Fla. 8924 with positive BLUP value (0.92) for *XY*, but negative value (− 0.43) for *SY*; however, they were absent in Fla. 8814 with negative BLUP value (− 0.17) for *XY*, but positive value (0.53) for *SY* (Additional file 2). A common association of chromosome 2 (*mID106*), which is approximately 7-Mbp away from one of molecular markers (TG608) for the mapping of *FW2.4*, was identified within a putative *Y* QTL identified in this study. Additionally, 11 unique association signals derived from either the filtered SNP set or regularization SNP set overlap with the linkage mapping (Additional file 6). Among them, most (seven out of eight) *SY*-associated signals are located predominantly on chromosomes 5 (three signals) and 11 (four signals). Two (*mID453* and *mID457*) of these four chromosome 11 *SY* signals were within the 5.3-Mbp interval determined by two different linkage mapping populations (populations A and D), and the interval is located < 1-Mbp upstream of the known fruit size/shape loci *FW11.3* [76] and *FASCIATED* [77].

Introgression of disease resistance, which is common and often a necessity for successful tomato cultivar development, impacts several horticultural traits including fruit size (weight) [78]. A signal (*mID566*) detected in the GWA mapping using the filtered SNP set was associated with *Y* (Additional file 6); it is located in the multi-megabases interval (approximately 4-Mbp) that carries the tomato spotted wilt virus-resistance *SW7* introgression [79].

### GEBVs for fresh-market tomato yields

**Fig. 3:**
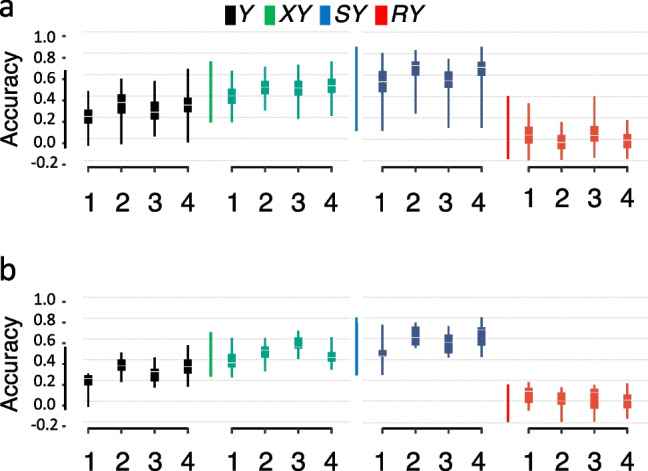
Testing and validation of prediction accuracies of genomic estimated breeding values (GEBVs) in fresh-market tomato yields. **a** Prediction accuracies of four different traits (*Y*, the total yield; *XY*, the yield of extra-large-sized fruit; *SY*, the yield of any fruit smaller than medium size; *RY*, the yield of red-colored fruit regardless of size) in 68 core inbred contemporary fresh-market tomatoes (inbred tomato set) are calculated using four different sets of SNPs and using an SVM-linear prediction model. Numbers 1 through 4 below each *X*-axis indicate four different sets of SNPs: 1, mapped SNP set (558 SNP sites); 2, regularization SNP set (364 SNP sites); 3, mapped/regularization SNP set (866 SNP sites); and 4, distributed SNP set (7266 SNP sites) (**a** and **b**). Colored rectangles indicate the 25th and 75th percentiles of the means of prediction accuracies; error bars indicate the ranges for the mean values (**a** and **b**). Prediction accuracies calculated using other models (rrBLUP, SVM-radial, SVM-poly, and random forest) can be found in Additional file 11. **b** Validation for prediction of *Y*, *XY*, *SY*, and *RY* traits using 420 F_2_ individual plants derived from four different F_2_ populations (F_2_ population set) and using SVM-linear. Plots are color-coded according to figure **a**

We generated GEBVs for the four traits investigated here in the inbred tomato set applying four different sets of SNPs (mapped SNP set, regularization SNP set, mapped/regularization SNP set, and distributed SNP set) and five different models (rrBLUP, SVM-linear, SVM-radial, SVM-poly, and random forest). In the training and testing using the inbred tomato set, all models showed higher accuracy for *XY* (the 25th and 75th percentiles of the means of prediction accuracies ranged from 0.4 to 0.6) and *SY* (the 25th and 75th percentiles of the means of prediction accuracies ranged from 0.5 to 0.8) regardless of SNP datasets tested as compared to that for *Y* and *RY* (Fig. 3a, Additional file 11). For *Y* prediction, one arm of range bars was close to 0 regardless of the SNP dataset or model. For *RY* accuracies, most of the 50th percentile of the means were close to 0. Overall, the means of prediction accuracies calculated using the regularization SNP set (364 SNPs) were compatible with those calculated using the distributed SNP set (7266 SNPs) and were slightly higher than those calculated using the mapped SNP set (558 SNPs). We chose the SVM-linear model to perform a validation experiment using the F_2_ population set (420 F_2_ individual plants derived from crosses between inbreds that were part of the inbred tomato set). After the SNP sets were trained on the inbred tomato set, prediction accuracies for traits on F_2_ individuals were calculated, and the mean accuracy for each trait was similar to that observed in the inbred tomato set (Fig. 3b).

## Discussion

In this study, we report two major new findings: (i) DNA sequence variant-trait associations for yields are evident from both eight previously mapped fruit size loci and 10 new association signals in the contemporary fresh-market tomato; (ii) the 25th and 75th percentiles of the means of prediction accuracies of different GEBVs calculation models range from 0.4 to 0.6 for *XY* trait and from 0.5 to 0.8 for *SY* trait, which are stratified by fruit size. The implications of these findings are discussed below.

### Objective assessment of tomato yield

In this study, a more objective assessment of fresh-market tomato yield was adopted by harvesting all fruits on a single harvesting date. Fresh-market tomato breeding programs routinely assess yield; however, a conventional fruit harvesting practice in the field is not amenable to an objective assessment of yield because most breeding programs manually harvest fruits based on individual personnel’s judgement of the fruit size and color in the field. Variations associated with fruit collection during manual harvest are further hindered by the presence of dense vegetative tissues and woody lateral branches (i.e., fruits hidden by leaves and entangled in ties), which is the typical morphology of fresh-market tomatoes with the support of stakes and ties at harvest. Therefore, the data from this study could aid tomato researchers by supporting an expansion of standardized phenotyping trials (discussion in Lee and Hutton [80]).

### Genome-wide empirical SNP filtering

Regularization simplifies overfit models and improves accuracy [46]; both L1 and L2 regularizations have been used for GWA mapping and GS in plants. The L1 regularization performs both shrinkage and variable selection to filter SNPs, and is used to map a putative, small effect plant height locus for this type of tomato [52]. In this study, the regularization SNP set was prepared after filtering genome-wide empirical SNPs using the L1 regularization to retain effective predictors with a smaller size; this provided an additional support for detecting significant GWA signals. The low percentage of significant SNP sites with or without the regularization methods (2 and 11% of significant SNPs retained respectively) could be attributed to the loss of SNP sites due to the multi-locus models controlling for multiple SNPs in the relatively narrow genomic region [81–83]. This low percentage of significant SNP sites consequently resulted in a large proportion of the association signals here became unique positions in either GWA mappings. We cannot rule out the possibility that potential true positive SNPs were lost after the filtering using the regularization methods [84, 85].

### Implications for tomato breeding

In this study, multiple previously known fruit size (weight)/shape loci including *FW2.2*, *FW3.2*, *LOCULE-NUMBER*, and *OVATE* were not detected for traits examined in this study (Minor allele frequencies of those loci < 0.06; Additional file 12). Therefore, the level of genetic contribution from those known loci is limited in the yield variation among the contemporary fresh-market tomatoes. Nonetheless, several significant associations overlap with fruit size (weight) loci previously identified as being involved in the domestication and improvement sweeps, especially *FW1.1* and *FW9.1* [39], indicating continued contribution for fruit yield in contemporary fresh-market tomato germplasm. We identified multiple SNP alleles with potential for improving the yield of fresh-market tomato. SNP alleles-associated with *XY* or *SY* could enable for selecting for high large-fruit yielding trait or selecting out high small-fruit yielding trait, respectively. The direction of effect of significant SNPs, following a distinct pattern especially between *XY* and *SY*, will further stimulate such selections. In addition to the eight SNP alleles identified at previously mapped fruit size (weight) loci (*FW1.1*, *FW8.1*, *FW9.1*, *FW9.2*, and *FW12.2*), 10 significant SNPs that do not overlap with previously mapped yield component loci could be used in molecular breeding. The 18 significant common association signals identified in this study represent plausible candidates for gene function.

We cannot rule out the possibility that causative genetic variations at yield loci are not SNPs. For example, the genetic variation at the *GLOBE* locus is an indel [10], although the locus was not associated with the examined yield variations (Additional file 13). Diverse types of genetic variations besides SNPs mediate a number of valuable plant traits [e.g., soybean cyst nematode resistance [86–88]]. Together with the results from this study, this suggests the use of further approaches to discover genetic variants influencing traits, such as studies analyzing diverse sequence variants (such as structural variants) together with SNPs [89]. In addition, diverse reference-level genomes (i.e., genomes finished to the compatible standard as the 1st released reference genome that is typically the best quality reference genome) might require the identification of the complete spectrum of genome-wide genetic variations in a given tomato because aligning fragments of re-sequenced reads to a single reference might lead to misinterpretation of variants and/or a failure to discover existing variants (such as the tomato resistance gene as reported in Lee [64]).

Artificial selection of traits through phenotype and/or genotype is the basis of all breeding programs. Substituting genotypic selection for phenotypic selection, especially where genetic correlations between the genotype and phenotype are high, is an increasingly important practice in contemporary breeding. Many inbred tomatoes used in this study have been commercialized (e.g., both inbred parental lines of Tasti-Lee F_1_, a fresh-market tomato cultivar currently in the U.S. market (e.g., Publix Super Markets, Inc.,) [90]) and transferred to public/commercial tomato research programs [91]. A cross between two contemporary fresh-market tomatoes (*S. lycopersicum* × *S. lycopersicum*) is the most common type of initial crosses in fresh-market tomato improvement programs. Therefore, the selection of SNPs associated with yield, especially marketable fruit yield (e.g., *XY*), could be highly beneficial for rapidly incorporating improved genotype combinations to achieve improved consumer-market-driven yields.

High yield of large fruits is a particularly important trait for fresh-market growers, especially in the U.S., because fresh-market tomato fruit can be sold in packages that meet a net standard weight and fruit-size requirements [3]. Therefore, most fresh-market tomato breeders have focused on that trait. Molecular marker-assisted selection or backcrossing focusing on major favorable traits (genes), such as disease resistance, are commonly employed in fresh-market tomato improvement programs [4]. However, most programs rely only on phenotypic selection for yield. In case of total yield and subsets of the total yield depending on specific fresh-market tomato markets, there are well-known challenges in measuring phenotypes that are sensitive to environmental effects or identifying a progeny with the highest breeding value. Furthermore, breeders/geneticists must simultaneously select for many favorable QTL, both major and minor effect QTL, for yield improvement. To the best of our knowledge, this is the first report to describe the significant DNA sequence-yield associations attributable to the genetic architecture of fresh-market tomato yield. Although the prediction accuracies of GEBVs using these associations were slightly lower than those using the genome wide SNPs, our study provides a framework for adopting GS for yield in the fresh-market tomato. Results (the heritability, collinearity, and prediction accuracies for traits examined here) demonstrate the potential for the application of GS for *XY* and/or against *SY*, which could be better practiced than that for *Y* or *RY*. Researchers can build GS models in which empirical associations are fit as fixed effects to maintain the characterized genetic architecture of consumer-market-driven specific subsets (e.g., *XY*) of the yield along with unidentified sequence variations responsible for target traits most likely located throughout the genome. Testing QTL effects and breeding values in appropriately structured populations with different filial generations (such as early segregating generation vs. fixed generation) is the first necessary step towards achieving their optimized models. Importantly, despite the reduction of SNP sites, the prediction accuracy of the different GEBV calculation models using the regularization SNP set was compatible for *XY* and *SY* traits, when it was compared with the accuracy of the distributed SNP set. This demonstrated a specific SNP set identified for prediction irrespective of GWA mapping may have a practical value; however, for some traits, the chosen regularization (e.g., LASSO or Bayesian LASSO) should be optimized to maximize computational efficiency.

For *Y* and *RY*, both linear (rrBLUP, SVM-linear) and non-linear (SVM-radial, SVM-poly, random forest) models exhibited low prediction accuracies, likely because of the low heritability for those traits (Similar cases are reported in rice [92]). Additionally, the phenotypic variations in *RY* could be strongly influenced by the environmental factors such as temperature, moisture. Therefore, achieving a fast genetic gain for *RY* remains a challenge. A high degree of positive correlation between *RY* and *XY* could provide clues to identify shared genetic variations between those traits.

### Beyond yield improvement

The positive effects from both previously known loci and new associations for yield imply an intriguing hypothesis about positive/negative allele effects for other important, but not yet fully investigated, traits in a contemporary fresh-market tomato germplasm. The presence of lower amounts of several flavor chemicals in modern commercial varieties compared to that in the older tomatoes was found in a comparison between the modern and older (such as heirloom, wild tomatoes) tomatoes [13]. However, it is unclear whether introduction of the phenotypic (flavor) variation found in wild tomatoes is necessary to improve the flavor of fresh-market tomato, because whether genetic loci responsible for lost flavor are non-existent, rare, or small in effect in the contemporary fresh-market tomato germplasm remain unknown in most cases. More importantly, the presence/absence of phenotypic/genotypic variance underlying much of the variation in fresh-market tomato flavor could be detectable by comparing fresh-market tomatoes with each other rather than through a comparison between the modern and old tomatoes. A preliminary fruit Brix evaluation showed a mean 4.5% Brix with a range of 3.9–6.4% in a group of fresh-market tomato germplasm used in this study (Additional file 14). Therefore, there could be unidentified genetic variance responsible for fresh-market tomato flavor. Making a new inter-species crossing [such as small-fruited wild tomato *Solanum pimpinellifolium* × large-fruited, domesticated tomato *S. lycopersicum* (or even a cross between two tomatoes from different market classes such as heirloom tomato and commercial fresh-market tomato)] is not often preferable for breeding and improvement programs because it inevitably compromises existing superior marketable traits (such as commercially acceptable yield potential, fruit quality including shape/firmness, and/or disease resistance). The general negative relationship observed between the fruit size and important horticultural traits including disease resistance [78] and sugar content [13] challenges such a cross. Therefore, future studies should focus on identifying the positive/negative effect alleles underlying flavor variation in the contemporary fresh-market tomato germplasm (breeding lines already selected for such marketable traits and genetic diversity). This could be immensely beneficial for rapidly incorporating good flavor into breeding backgrounds (similar to a disease resistant tomato breeding material [64, 65]).

## Conclusions

Fruit yield is the most important trait for most fruit crops. This is the first study that reports the DNA sequence-trait associations for the total fresh-market tomato yield and subsets of the yield responsible for different fruit markets. The genetic architecture of tomato yield characterized in this study will be an important resource for future tomato research, including validating the association signals identified in this study and using these results to develop effective tools such as GS for improving the efficiency and speed of breeding for the marketable fruit yield. In addition, the results from this study emphasize the value of expanding this research to other important but yet poorly understood fresh-market tomato traits.

## Methods

### Plant material

The collected 68 core contemporary fresh-market tomatoes (hereafter referred to as inbred tomato set) met four requirements: i) a pool of released elite inbred cultivars or inbred breeding lines that have been selected for marketable traits including yield and genetic diversity (many with commercialization and public/commercial production pedigrees [91]), ii) those included in a strategic planning of current breeding effort, iii) those with maximum diversity based on field performance, while avoiding shared breeding pedigree as much as possible, and iv) those that are not plant introductions (PIs) originating outside the U.S. Seeds of these tomatoes were obtained from UF/IFAS, 2022. In addition, four biparental F_2_ populations derived from eight different contemporary fresh-market tomatoes that were part of the inbred tomato set were developed in the T.G.L. laboratory [hereafter, F_2_ population set; F_2_ population A (a cross Fla. 7946 × Fla. 7776; an F_1_ hybrid is released and commercialized by Scott et al. [93]), B (Fla. 7907B × Fla. 8059; an F_1_ hybrid is released and commercialized by Scott et al. [90]), C (Fla. 8249 × Fla. 8124C), and D (Fla. 7771 × Fla. 7060) (104, 104, 105, or 107 plants per each F_2_ population, respectively)].

### Phenotypic data collection and analysis

Field trials of the inbred tomato set were conducted during three consecutive years, 2019, 2020, and 2021 at the UF Gulf Coast Research and Education Center (GCREC; Wimauma, FL, USA), where these tomatoes were originally bred, as described previously (‘Field trial’ section of Lee et al. [6]). For the first, second, and third growing cycles, seed sowing in the greenhouse and fruit harvest were performed on August 5 and December 20, August 3 and December 23, and July 16 and December 2, respectively. Simultaneously, the F_2_ population set was grown in a field plot neighboring the inbred tomato set in 2020. For both the inbred tomato set and F_2_ population set, fruit collection and yield (kg per plant) evaluations were performed, as described in our previous study (‘Field trial’ and ‘Greenhouse trial’ sections of Lee et al. [6]). Briefly, all fruits with visually identifiable size (approximately > 0.5 cm in diameter) developed in each season were harvested on a single harvesting date, regardless of fruit size, quality (e.g., irrespective of whether the fruits had defects such as cracks), color, or flowering clusters bearing fruits; Fruits were sorted by size [3]; and by color into two classes, green (*G*; USDA color classification ‘Green’ [94]) and red (*R*; Fig. S10 in Lee et al. [6]; USDA color classification ‘Breakers’ [94]). For each of the four different traits, the fruit yield (kg per plant) stratified by size or color was calculated [(i) *Y* regardless of size or color, (ii) *XY* regardless of color, (iii) *SY* regardless of color, (iv) *RY* regardless of size]. A normality test was performed for each trait data set from each year using Anderson-Darling at a significance level of 0.05. Phenotypic datasets from three seasons were used to calculate the best linear unbiased prediction (BLUP) values of each tomato using a model, lmer(phenotype ~ (1|genotype) + (1|Year) + (1|genotype:Year)), implemented in the R package ‘lme4’ (version 1.1–28) [95] (Additional file 2). The narrow sense heritability for each trait was estimated on a line means basis using ‘lm4’. Pearson’s correlation among the phenotypic traits were estimated from BLUPs using the R package ‘Performance Analytics’ (version 2.0.4). Multi-collinearity between the traits was estimated using the R package ‘mcvis’ (version 1.0.8) [96].

To create a phenotypic dataset for the prediction of GEBVs, 15 and 20 additional traits (i.e., yield components stratified by the fruit number, size, and color) were evaluated for the inbred tomato set and F_2_ population set, respectively, following the same basic procedures used for *Y*, *XY*, *SY*, and *RY* traits and our previous study [80] (Additional file 15). Nine traits evaluated for both sets are as follows: *FN*, the total number of fruits per plant; *MLXFN*, the number of medium-sized or larger fruits; *MLXRFN*, the number of medium-sized or larger red fruits; *MLXRY*, the yield of medium-sized or larger red fruits; *MLXY*, the yield of medium-sized or larger fruits; *RFN*, the number of red fruit; *SFN*, the number of any fruit smaller than medium size; *SRY*, the yield of any red fruit smaller than medium size; and *XFN*, the number of extra-large fruit. Six traits evaluated for the inbred tomato set only are as follows: *LFN*, the number of large-size fruit; *LY*, the yield of large-size fruit; *MFN*, the number of medium-size fruit; *MY*, the yield of medium-size fruit; *SW*, the average weight of any fruit smaller than medium size; and *W*, the average fruit weight per plant. Eleven traits evaluated for the F_2_ population set only are as follows: *GFN*, the number of green fruit; *GY*, the yield of green fruit per plant; *MLXGFN*, the number of medium-sized or larger green fruits; *MLXGY*, the yield of medium-sized or larger green fruits; *SGFN*, the number of any green fruit smaller than medium size; *SGY*, the yield of any green fruit smaller than medium size; *SRFN*, the number of any red fruit smaller than medium size; *XGFN*, the number of extra-large-sized green fruit; *XGY*, the yield of extra-large-sized green fruit; *XRFN*, the number of extra-large-sized red fruit; *XRY*, the yield of extra-large-sized red fruit. The BLUP values were calculated for each of the traits in the inbred tomato set as described above.

### Whole-genome resequencing and SNP calling

We performed WGS for 425 plants (five core tomatoes and 420 F_2_ plants of F_2_ population set). All sequence reads were created using the same technical conditions as follows: i) a single plant of each tomato line used for DNA extraction, ii) PCR-free 350-bp library preparation, iii) paired-end (2 × 150-bp) sequencing using Illumina NovaSeq technology, and iv) average sequence data of 23 Gb per core tomato (approximately 30 × genome coverage of each sample) or 5 Gb per F_2_ plant. For the raw reads, the quality controls were as follows: i) all positions having average Phred quality scores 30 or higher, ii) read without adapter sequence(s), and iii) read with ambiguous nucleotides (i.e., Ns) < 10%. In addition to the sequence reads we generated in this study, 63 raw read datasets of contemporary U.S. fresh-market tomatoes, which were generated by using the same technical conditions and quality control as described here, were obtained from our previous studies [23, 97].

SNP detection was performed as described in our previous study [97]. Once SNP detection was completed for each plant, the SNP calls supported by fewer than three reads were removed. Two separate SNP datasets were prepared for GWA mapping and linkage mapping. For GWA mapping, SNP sites were further filtered based on minor allele frequency (MAF) > 0.07, Hardy-Weinberg equilibrium of 1.0 × 10^− 5^, and missing genotype rate < 5% using PLINK (version 1.90b3) [98], resulting in a final set of 301,536 SNPs (hereafter, filtered SNP set). For linkage mapping, SNP sites were further filtered using the following settings: i) < 50% missing genotype codes and ii) the first SNP site per 10-k-base pair (kbp) interval across the genome (hereafter, linkage SNP set).

To validate heterozygous alleles at SNP sites, we tested 16 SNP sites through phasing analysis using informative bases derived from paired-end reads from single molecules similar to that used for repeat subunit assembly in a previous study (‘Repeat subunit assembly and type definitions’ section of Lee et al. [87]). First, we configured mapped, paired reads that possessed variants including those from heterozygous alleles. Two variants that reside on the same read or the corresponding mate in paired-end reads were considered to have originated from the same molecule; and therefore, be used to define a heterogeneity. Second, genomic sequences that are similar to the chosen flanking regions of the 16 SNPs were identified using nucleotide blast search (https://blast.ncbi.nlm.nih.gov/Blast.cgi; query, 325 bp sequence of SL4.0 reference genome assembly, which flanks a SNP position; subject, SL4.0 reference genome assembly). We examined sequence variant(s) that were on the top two Blast hits, with respect to Identity (%). For eight out of the 16 SNP sites, the top two Blast hits carry each of two alleles at each SNP site (Additional files 16 and 17). However, none of the sequence variant(s) at the SNP sites that reside on the 2nd top Blast hit matched the phased variants, strongly suggesting apparent heterozygosity at those 16 SNP sites.

### Population genetics analysis

VCFtools (version 0.1.15) [99] was used to calculate nucleotide diversity (*π*) (a 10-kbp window with 5-kbp increments, while accepting 20% missing genotype codes) and inbreeding coefficient (*F*) on the filtered SNP set. Principal component analysis (PCA) was performed using PLINK (version 1.90b3) [98] on the same SNP set.

### Genome-wide association mapping

In addition to the filtered SNP set described above, another SNP set (referred to as regularization SNP set), was prepared; two different SNP sets, filtered SNP set and regularization SNP set, were used for the GWA mapping. To develop the regularization SNP set, the effect of SNPs for each of four traits (*Y*, *XY*, *SY*, and *RY*) were estimated using both LASSO and MCP regularization methods as described in our previous study [52]. The SNPs with non-zero effect for at least one of these traits in either of the methods resulted in a set of 364 SNP sites (regularization SNP set). For each SNP set, we performed six multi-locus random-SNP-effect mixed linear model using the R package mrMLM with default parameters (version 4.0.2) [83]. The Balding Nichols kinship matrix computed using EMMAX (version 20,100,307) [100] and the first 10 PCs identified using the R function prcomp() were included as covariates to control population structure and individual relatedness, similar to that in the previous fresh-market tomato GWA study [23]. In addition, we used one-way ANOVA in conjunction with a two-tailed Tukey’s HSD multiple comparison test to determine the association between the BLUP values for yields (*Y*, *XY*, *SY*, and *RY*) and the GWA mappings-derived individual significant association signals, at a significance of *p* < 0.05. The genetic position (cM) of association signals were estimated based on a genetic map for the U.S. fresh-market tomato [97]. The genetic distance between two SNP markers that were flanking an association signal in the genetic map were averaged.

### QTL linkage mapping

After calculating the genotype probabilities using the R function calc.genoprob(), a QTL mapping function stepwiseqtl() [101] of the R/qtl package was used to map QTL (LOD > 3.5) of 28 traits in each of the four F_2_ populations (F_2_ population set). A confidence interval length was calculated using the R function qtl.length() in the R package ‘qtldesign’ (version 0.941) [102]; the linkage SNP set was used as genotypic datasets.

### Generation of GEBVs

Four different SNP datasets [i) a set of combined significant SNP sites, which were identified by either the GWA mapping of 28 traits using the filtered SNP set or QTL linkage mapping (hereafter, mapped SNP set; 558 SNP sites; Additional file 18), ii) regularization SNP set (364 SNP sites), iii) a combined SNP sites of both mapped SNP set and regularization SNP set (mapped/regularization SNP set; 866 SNP sites), and iv) the first SNP site per 10-kbp interval across the tomato genome (distributed SNP set; 7266 SNP sites)] were used. To estimate the GEBVs for each trait, five different models were used: i) ridge regression using an R function mixed.solve() in the R package rrBLUP (version 4.6.1) [103], ii) Radial, non-radial, and polynomial kernel in support vector machine (SVM) implemented in the R package ‘caret’ (version 6.0–90) [104], and iii) random forest, a tree-based model implemented in the ‘caret’. To assess prediction accuracy, we performed 5-fold cross-validation. One of the five folds of 68 tomatoes in the inbred tomato set served as the validation fold, and the other four folds served as the training folds. Each combination of training set and algorithm was averaged over 100 iterations for accuracy estimate. The mean squared errors (MSEs) of the models were assessed for model reliability (Additional file 19). GEBVs of the inbred tomato set was validated using the F_2_ population set (420 F_2_ individual plants). The whole 68 tomatoes in the inbred tomato set was used as a training set.

## Supplementary Information

**Additional file 1.**

**Additional file 2.**

**Additional file 3.**

**Additional file 4.**

**Additional file 5.**

**Additional file 6.**

**Additional file 7.**

**Additional file 8.**

**Additional file 9.**

**Additional file 10.**

**Additional file 11.**

**Additional file 12.**

**Additional file 13.**

**Additional file 14.**

**Additional file 15.**

**Additional file 16.**

**Additional file 17.**

**Additional file 18.**

**Additional file 19.**

## Data Availability

The datasets used and/or analyzed during the current study are available from the corresponding author on reasonable request.
