# Supplementary material for: Genetic architecture of fresh-market tomato yield
Source: BMC Plant Biol. 2023 Jan 9;23:18. doi: 10.1186/s12870-022-04018-5 (PMC9827693; doi:10.1186/s12870-022-04018-5)
Supplement: Supplementary file 3 — Additional file 3. [file 12870_2022_4018_MOESM3_ESM.pdf]

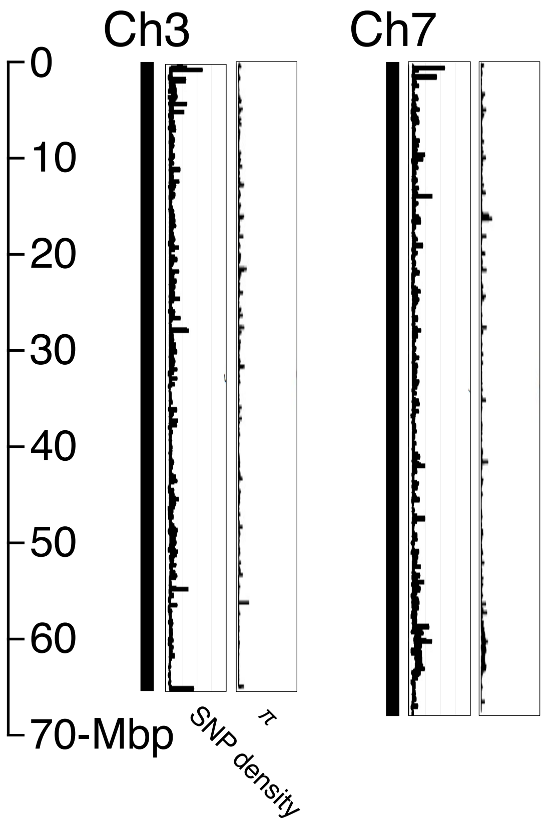

Additional file 3: Supplementary Fig. 1 (pdf). Single nucleotide polymorphism (SNP) diversity of chromosomes 3 and 7. SNP density across the genome (left panel) and nucleotide diversity (π) (right). For each chromosome, SNP density (the range of the *Y*-axis 0 to 400 SNPs per 10-kbp) and nucleotide diversity (π) (the range of the *Y*-axis 0 to 0.08) plots are provided.
