# Supplementary material for: Genetic architecture of fresh-market tomato yield
Source: BMC Plant Biol. 2023 Jan 9;23:18. doi: 10.1186/s12870-022-04018-5 (PMC9827693; doi:10.1186/s12870-022-04018-5)
Supplement: Supplementary file 4 — Additional file 4. [file 12870_2022_4018_MOESM4_ESM.pdf]

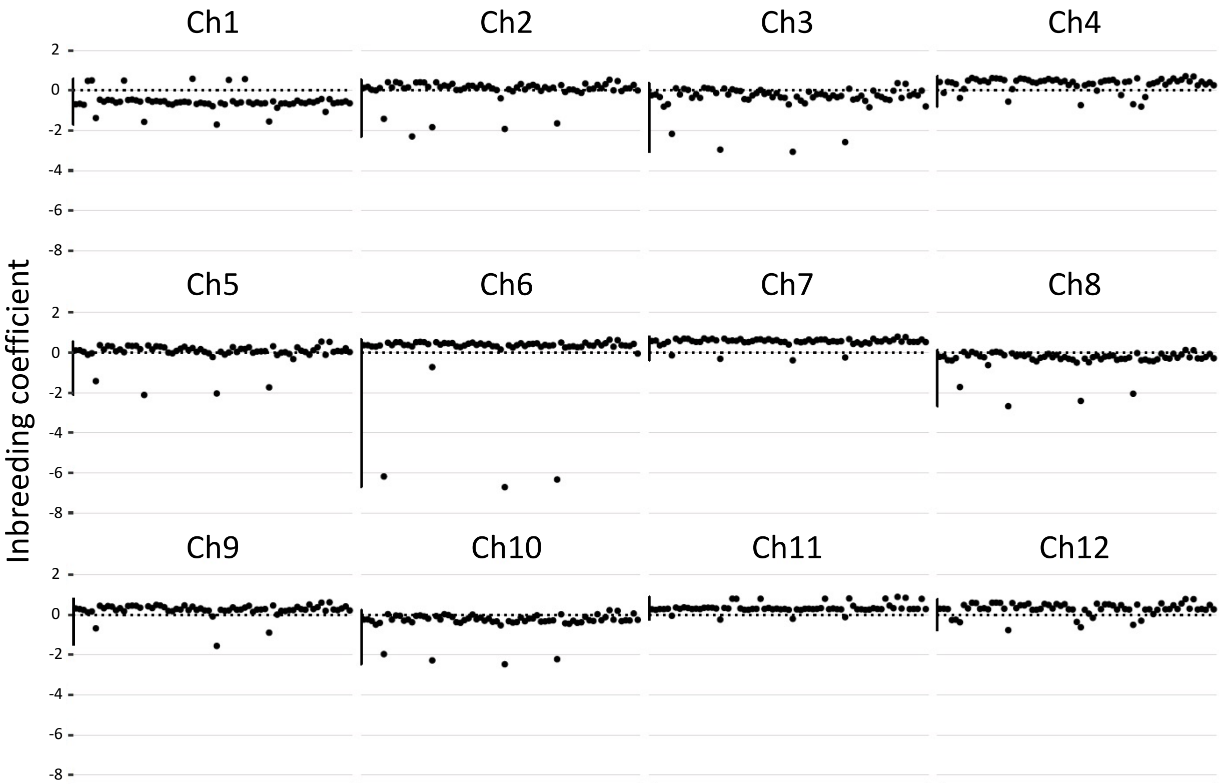

Additional file 4: Supplementary Fig. 2 (pdf). Inbreeding coefficient (*F*) across the tomato genome. Numbers above each *X*-axis indicate chromosomes.
