# Supplementary material for: Genetic architecture of fresh-market tomato yield
Source: BMC Plant Biol. 2023 Jan 9;23:18. doi: 10.1186/s12870-022-04018-5 (PMC9827693; doi:10.1186/s12870-022-04018-5)
Supplement: Supplementary file 5 — Additional file 5. [file 12870_2022_4018_MOESM5_ESM.pdf]

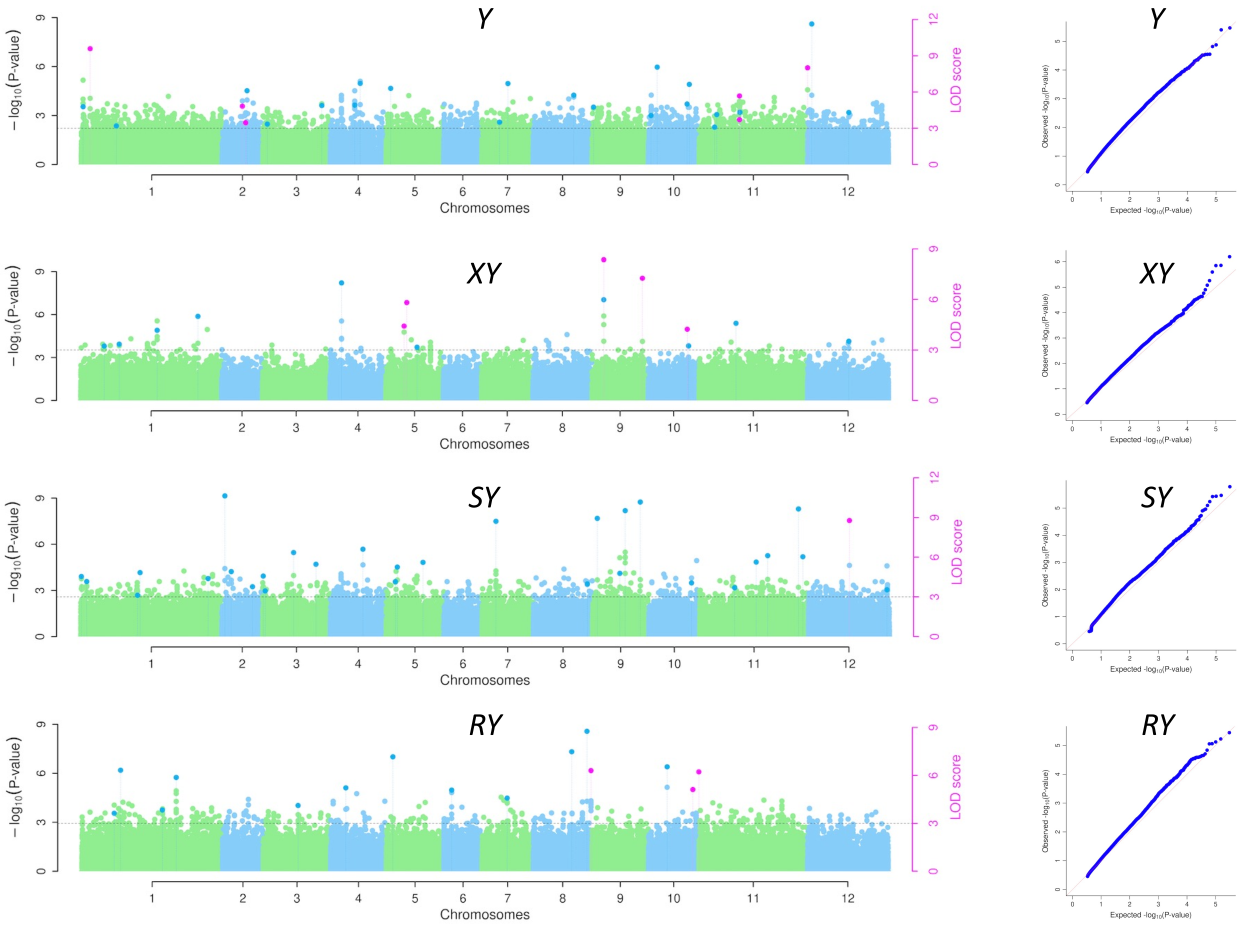

Additional file 5: Supplementary Fig. 3 (pdf). GWA mapping results using the filtered SNP set. Manhattan plots of SNPs associated with traits (left panel) and Quantile-quantile (QQ) plots for association mapping (right). Gray dashed lines in Manhattan plots indicate the default critical logarithm of the odds (LOD) score threshold. Red lines in QQ plots signify concordance between observed and expected associations.
