# Supplementary material for: Genetic architecture of fresh-market tomato yield
Source: BMC Plant Biol. 2023 Jan 9;23:18. doi: 10.1186/s12870-022-04018-5 (PMC9827693; doi:10.1186/s12870-022-04018-5)
Supplement: Supplementary file 8 — Additional file 8. [file 12870_2022_4018_MOESM8_ESM.pdf]

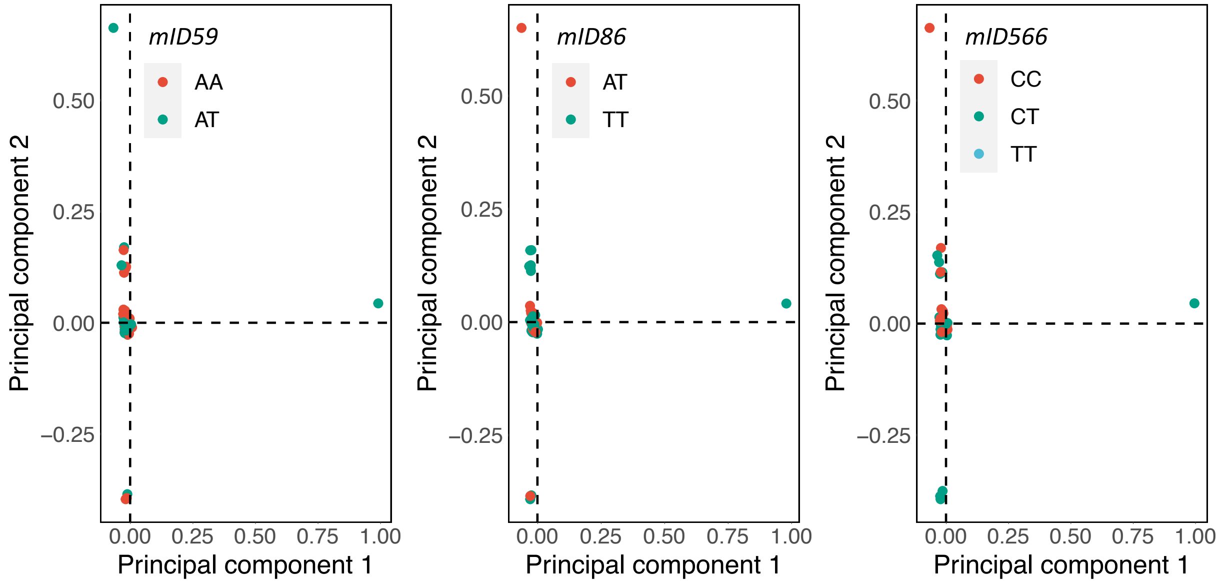

Additional file 8: Supplementary Fig. 5 (pdf). Principal component analysis (PCA) plots color-coded according to allele types. For the PCA plot, the first two principal components of SNP variation of the inbred tomato set is obtained from Fig. 1d. Three positions of association signals (one from significant common associations and two from the genome-wide association (GWA) mapping using the filtered SNP set) are displayed.
