# Supplementary material for: Genetic architecture of fresh-market tomato yield
Source: BMC Plant Biol. 2023 Jan 9;23:18. doi: 10.1186/s12870-022-04018-5 (PMC9827693; doi:10.1186/s12870-022-04018-5)
Supplement: Supplementary file 9 — Additional file 9. [file 12870_2022_4018_MOESM9_ESM.pdf]

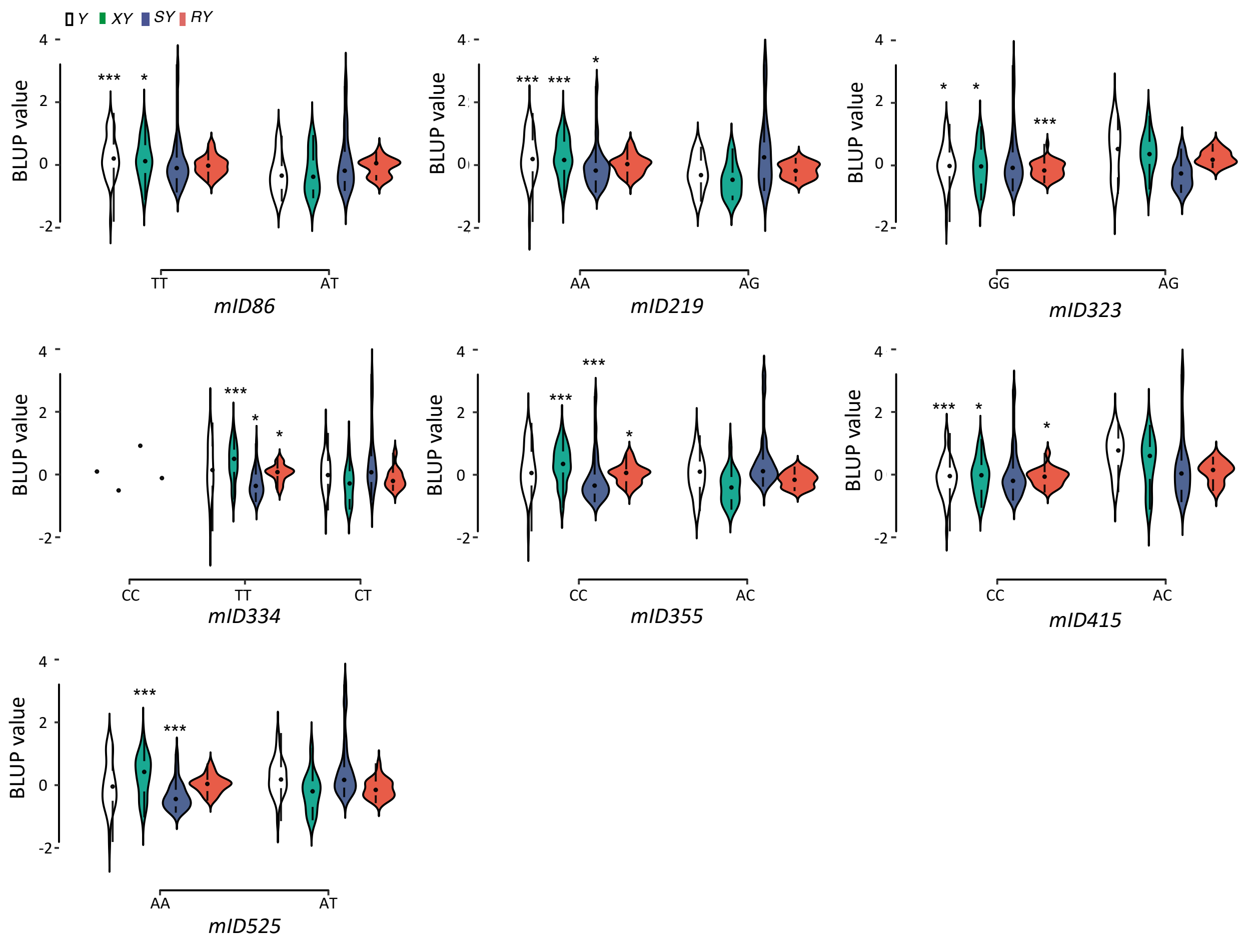

Additional file 9: Supplementary Fig. 6 (pdf). Association between the BLUP values for yields (*Y*, the total yield; *XY*, the yield of extra-large-sized fruit; *SY*, the yield of any fruit smaller than medium size; *RY*, the yield of red-colored fruit regardless of size) and the individual significant association signals, which is determined by ANOVA; statistically significance $^{*}p < 0.05$, $^{***}p < 0.001$.
