# Supplementary material for: Genetic architecture of fresh-market tomato yield
Source: BMC Plant Biol. 2023 Jan 9;23:18. doi: 10.1186/s12870-022-04018-5 (PMC9827693; doi:10.1186/s12870-022-04018-5)
Supplement: Supplementary file 10 — Additional file 10. [file 12870_2022_4018_MOESM10_ESM.pdf]

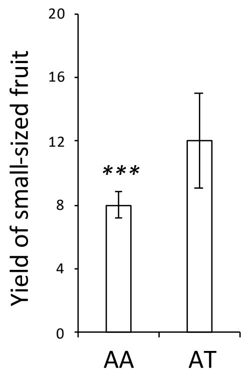

Additional file 10: Supplementary Fig. 7 (pdf). Contributions of different alleles at *mID525* for the yield of small-sized fruit (*SY*) is validated in a segregating F_2 population D. Error bars indicate 95% confidence intervals; statistically significance $^{***}p < 0.001$ by a two-tailed *t*-test.
