# Supplementary material for: Genetic architecture of fresh-market tomato yield
Source: BMC Plant Biol. 2023 Jan 9;23:18. doi: 10.1186/s12870-022-04018-5 (PMC9827693; doi:10.1186/s12870-022-04018-5)
Supplement: Supplementary file 11 — Additional file 11. [file 12870_2022_4018_MOESM11_ESM.pdf]

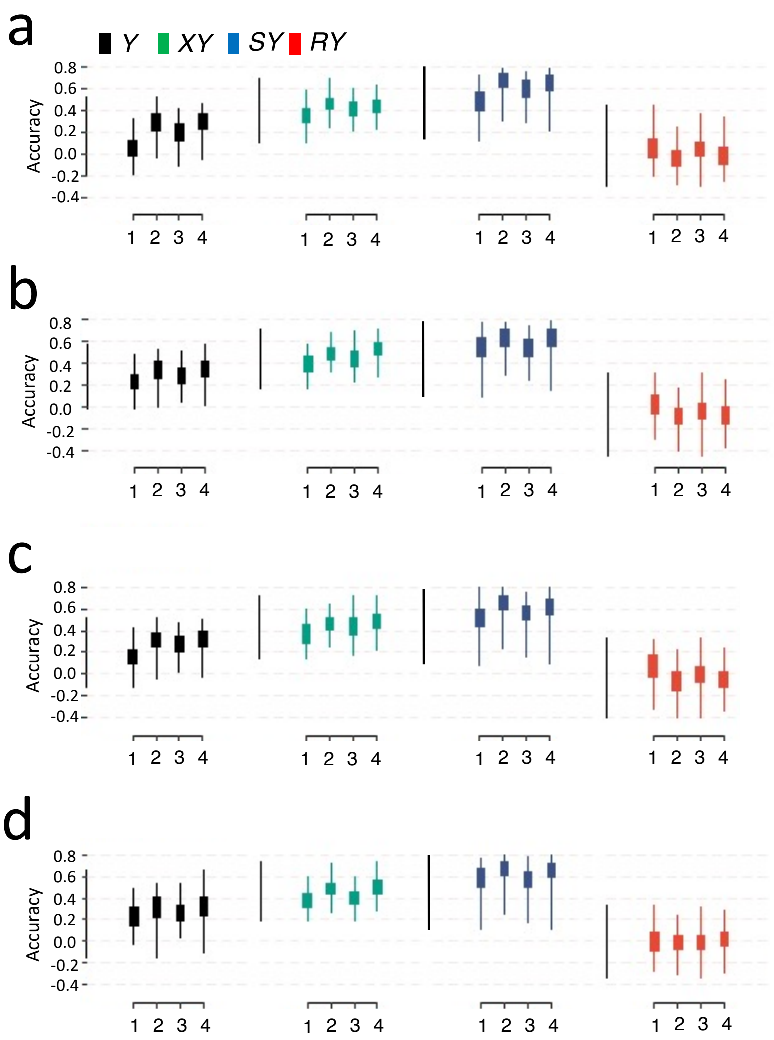

Additional file 11: Supplementary Fig. 8 (pdf). Testing of prediction accuracies of genomic estimated breeding values (GEBVs) in fresh-market tomato yields. Prediction accuracies of four different traits (*Y*, the total yield; *XY*, the yield of extra-large-sized fruit; *SY*, the yield of any fruit smaller than medium size; *RY*, the yield of red-colored fruit) in the inbred tomato set are calculated using four different sets of SNPs and using rrBLUP (**a**), SYM-radial (**b**), SVM-poly (**c**), and random forest (**d**). Numbers 1 through 4 below each *X*-axis indicate four different sets of SNPs: 1, mapped SNP set; 2, regularization SNP set; 3, mapped/regularization SNP set; and 4, distributed SNP set (**a** through **d**). Colored rectangles indicate the 25th and 75th percentiles of the means of prediction accuracies; error bars indicate the ranges for the mean values.
