# Supplementary material for: Genetic architecture of fresh-market tomato yield
Source: BMC Plant Biol. 2023 Jan 9;23:18. doi: 10.1186/s12870-022-04018-5 (PMC9827693; doi:10.1186/s12870-022-04018-5)
Supplement: Supplementary file 12 — Additional file 12. [file 12870_2022_4018_MOESM12_ESM.pdf]

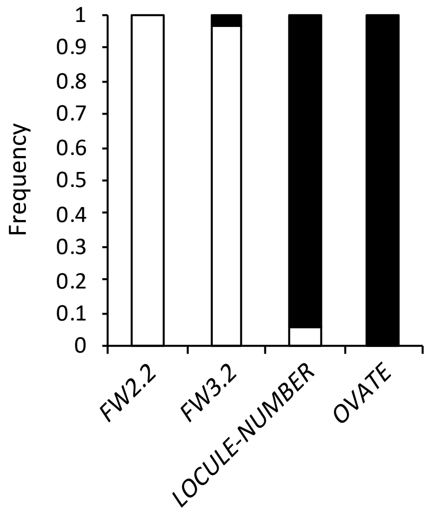

Additional file 12: Supplementary Fig. 9 (pdf). Allele distribution of four previously mapped fruit size (weight)/shape loci. The open and filled bars indicate the reference and alternate alleles, respectively. The tomato (Heinz 1706) genome version SL4.0 [63] was used as the reference; Heinz 1706 is a fully sequenced reference domesticated tomato.
