# Supplementary material for: Genetic architecture of fresh-market tomato yield
Source: BMC Plant Biol. 2023 Jan 9;23:18. doi: 10.1186/s12870-022-04018-5 (PMC9827693; doi:10.1186/s12870-022-04018-5)
Supplement: Supplementary file 13 — Additional file 13. [file 12870_2022_4018_MOESM13_ESM.pdf]

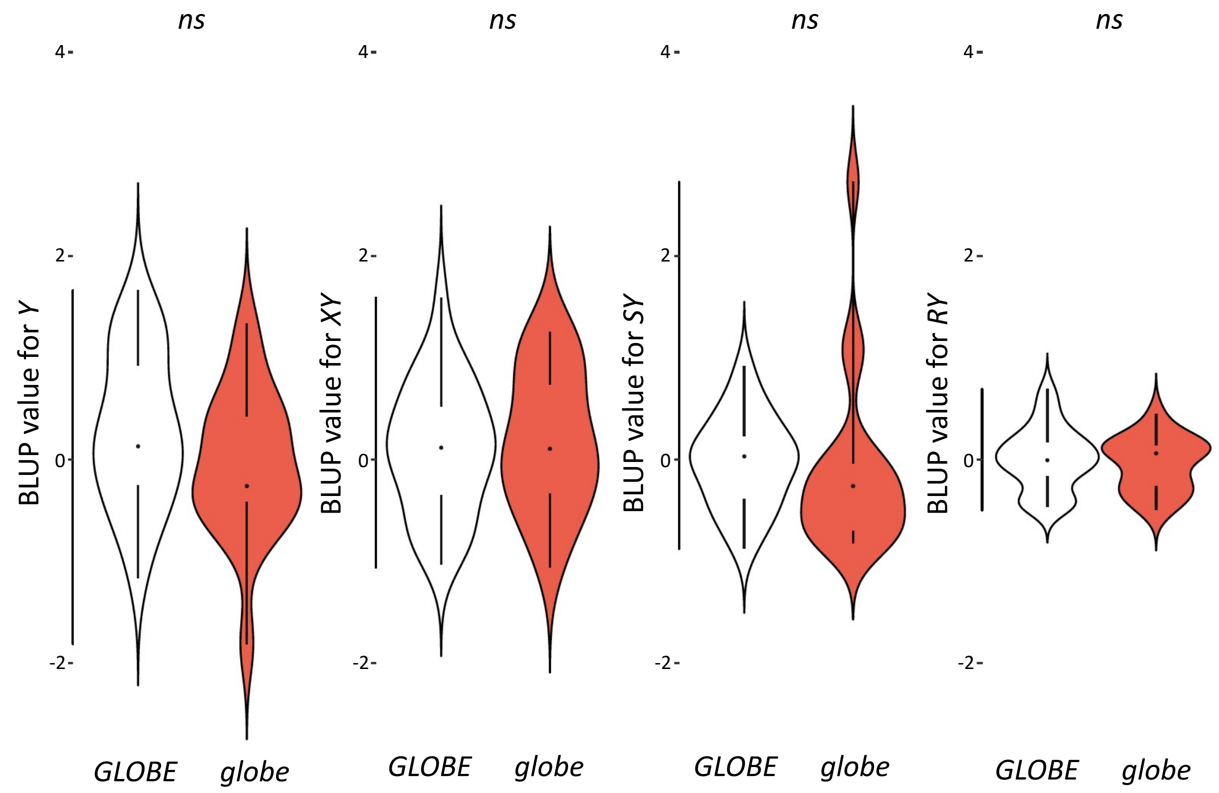

Additional file 13: Supplementary Fig. 10 (pdf). Phenotypic contribution of the *GLOBE* gene in the inbred tomato set. *GLOBE* and *globe* indicate two different alleles at the *GLOBE* locus. *ns* indicates no significant difference (ANOVA at $p > 0.05$).
