# Supplementary material for: Genetic architecture of fresh-market tomato yield
Source: BMC Plant Biol. 2023 Jan 9;23:18. doi: 10.1186/s12870-022-04018-5 (PMC9827693; doi:10.1186/s12870-022-04018-5)
Supplement: Supplementary file 15 — Additional file 15. [file 12870_2022_4018_MOESM15_ESM.pdf]

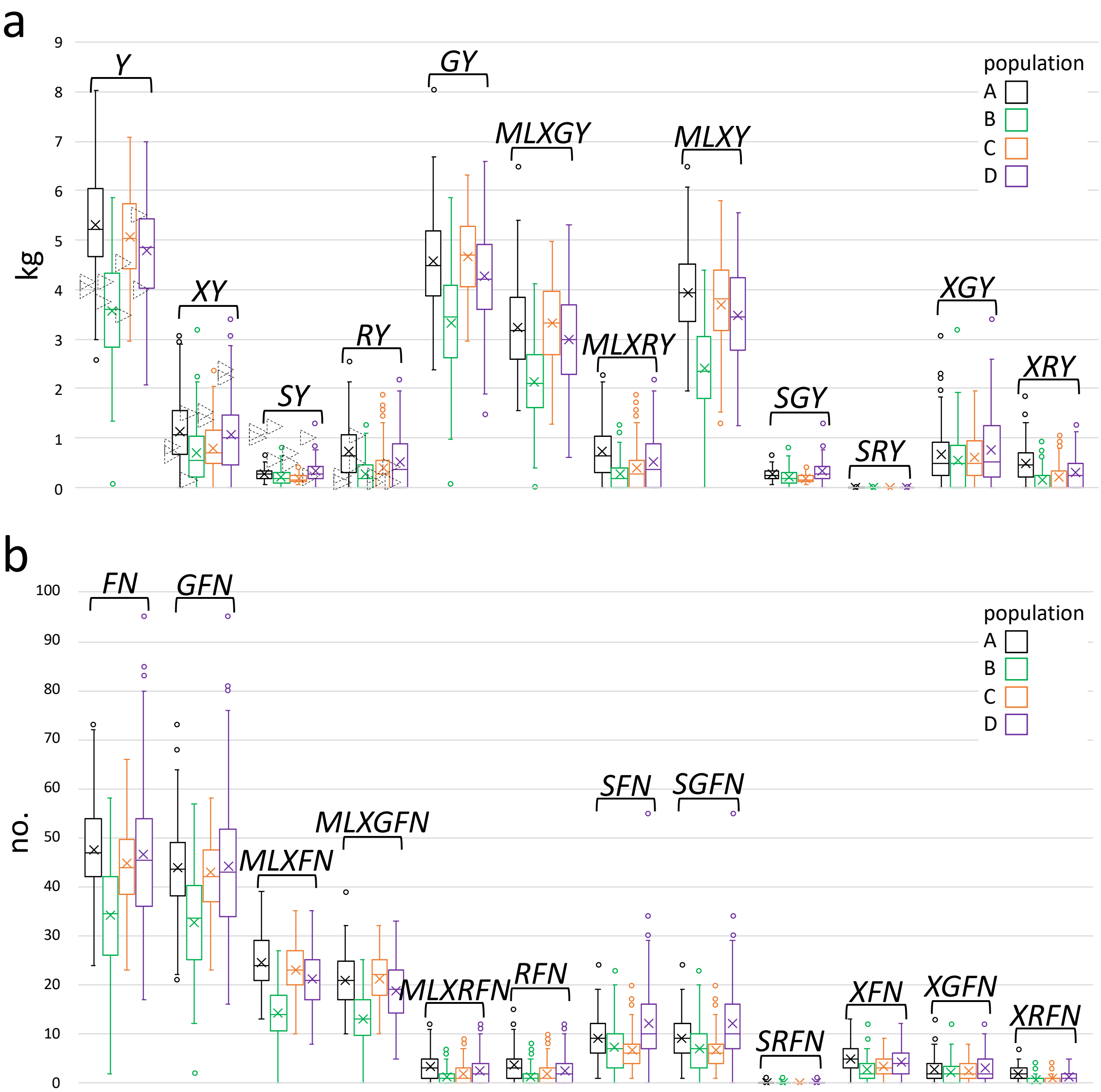

Additional file 15: Supplementary Fig. 11 (pdf). Distribution of phenotypic values in the F_2 population set. **a**. 12 yield traits (*Y*, the total yield; *XY*, the yield of extra-large-sized fruit; *SY*, the yield of any fruit smaller than medium size; *RY*, the yield of red-colored fruit regardless of size; *GY*, the yield of green fruit per plant; *MLXGY*, the yield of medium-sized or larger green fruits; *MLXRY*, the yield of medium-sized or larger red fruits; *MLXY*, the yield of medium-sized or larger fruits; *SGY*, the yield of any green fruit smaller than medium size; *SRY*, the yield of any red fruit smaller than medium size; *XGY*, the yield of extra-large-sized green fruit; *XRY*, the yield of extra-large-sized red fruit). Dashed arrows indicate

phenotypic values of parental lines. Colored rectangles indicate the interquartile range (25th and 75th quartiles), and the horizontal line and × symbols indicate the median and mean values, respectively (**a** and **b**). The whiskers indicate a range between 1.5 times the interquartile range below the 1st quartile or 1.5 times the interquartile range above the 3rd quartile. The upper and lower whiskers indicate the largest and smallest values. The dots represent outlying values. **b**. 12 fruit number traits (*FN*, the total number of fruits per plant; *GFN*, the number of green fruit; *MLXFN*, the number of medium-sized or larger fruits; *MLXGFN*, the number of medium-sized or larger green fruits; *MLXRFN*, the number of medium-sized or larger red fruits; *RFN*, the number of red fruit; *SFN*, the number of any fruit smaller than medium size; *SGFN*, the number of any green fruit smaller than medium size; *SRFN*, the number of any red fruit smaller than medium size; *XFN*, the number of extra-large fruit; *XGFN*, the number of extra-large-sized green fruit; *XRFN*, the number of extra-large-sized red fruit).
