# Supplementary material for: Genetic architecture of fresh-market tomato yield
Source: BMC Plant Biol. 2023 Jan 9;23:18. doi: 10.1186/s12870-022-04018-5 (PMC9827693; doi:10.1186/s12870-022-04018-5)
Supplement: Supplementary file 16 — Additional file 16. [file 12870_2022_4018_MOESM16_ESM.pdf]

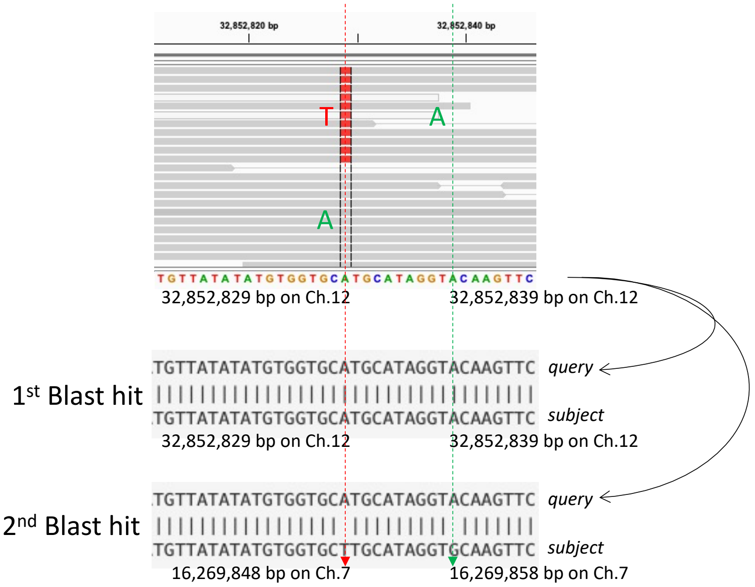

Additional file 16: Supplementary Fig. 12 (pdf). Validation of heterozygous alleles at SNP sites. **a**. Whole genome sequencing (WGS) alignment view. A heterozygous allele from a tomato inbred line Fla. 8814 at 32,852,829 bp (red arrow) on chromosome 12 is displayed in the Integrative Genomics Viewer (IGV) window. **b**. Nucleotide blast search used to validate the presence or absence of sequence variant(s) that reside on the single molecule. The top Blast hit (Identity 323/323) identifies a genomic sequence flanking the original SNP position at 32,852,829 bp on chromosome 12 (red vertical arrow). The 2nd Blast hit (Identity 309/323) identifies a genomic sequence on chromosome 7, which carries a G residue at the 16, 269, 858 bp on chromosome 7 where an additional single nucleotide mismatch exists between the subject and the query (green vertical arrow). See Additional file 17 for the complete information of 16 SNP sites validated.
